# The infant gut microbiota: in pursuit of non-protein nitrogen

**DOI:** 10.1080/19490976.2023.2211917

**Published:** 2023-05-24

**Authors:** Patrick Schimmel, Bernd Stahl, Jan Knol, Clara Belzer

**Affiliations:** a Department of Agrotechnology and Food Sciences, Wageningen University & Research Center, Laboratory of Microbiology, Wageningen, The Netherlands; b Danone Nutricia Research, Utrecht, The Netherlands; c Department of Chemical Biology & Drug Discovery, Utrecht Institute for Pharmaceutical Sciences, Utrecht University, Utrecht, The Netherlands

**Keywords:** Human, milk, nitrogen, breastfeeding, infant, gut, bacterial, metabolism, microbiome, health

## Abstract

Diet shapes our gut microbiome from the day we are born. The contribution of dietary non-protein nitrogen to normal and healthy nitrogen cycling in the infant gut is scarcely described. Herein, we review in vitro and in vivo findings that show the impact of Human Milk Nitrogen (HMN) on the gut microbiota that colonizes the gut in early human life. We describe that several non-protein nitrogen sources, that include creatine, creatinine, urea, polyamines and free amino acids, are key in establishing the bifidobacterium-dominated microbiome and thus are bifidogenic. Furthermore, several parts of HMN-related metabolism are associated with a healthy infant gut and commensal microbiota. We illustrate an overlap and great diversity in accessibility of HMN by large parts of the infant gut microbiota. This review nonetheless shows the importance of research on HMN and its effects on the activity and composition of the infant gut microbiota and its potential effect on early life infant health.

## Introduction

The human gut contains a microbial cell population, or microbiome, that through its presence and activity supports human immunology and prevents disease.^1^ This microbial support in our gut starts immediately after birth and is efficiently supported by human milk (HM).^2,3^ Early life provides a unique opportunity to study the effect of diet on microbiota. Even though the process of defining a healthy infant microbiota is ongoing, we understand that breastfeeding promotes a *Bifidobacterium*-dominated microbiome in healthy infants.^3,4^ However, variations in microbiota composition, geographically and inter-individually are high.^2,5^ Nonetheless, breastfed infants are considered a low-risk group for many health threats,^6,7^ thus making breastfeeding the golden standard for infant feeding. This guided research toward identifying prebiotics in HM that promote a beneficial microbiota. Fundamentally, to understand metabolism in the breastfed infant’s gut and more applicably to improve infant feeding alternatives. Therefore, a recent research focus was the bacterial acquisition of carbon in the infant gut. Human Milk Oligosaccharides (HMOs), a dominant carbon source in HM, are selectively utilized by *Bifidobacterium* spp. and are therefore promoting *Bifidobacterium* spp. in the infant gut environment.^8–10^ HMOs are not the only nutrient class in HM, and the bacteria have likely adapted to other molecules and nutrients present. Moreover, nitrogen is a fundamental need for bacteria to survive in any environment. This suggests that implementing oligosaccharides in the infant diet alone will not lead to a sufficient resemblance to a breastfed microbiota.^5,11^

An equally crucial pinnacle of survival for bacteria in the infant gut is not well documented, namely the acquisition of nitrogen. Meanwhile, research shows that microbial communities are profoundly affected by nitrogen availability.^12,13^ Breast milk is a complex bio-fluid and holds a thorough co-evolution orchestrated nitrogen content. While containing relatively low amounts of protein compared to the diet in later life, it has various other sources of nitrogen including secretory waste products of human metabolism, amino acids (AAs), polyamines and vitamins.^6,14–23^ The evolution of HM and a co-evolving microbiome makes it likely that the microbiota of the breastfed infant is adapted to low protein concentrations is utilizing this specific nitrogen supply to gain a competitive advantage. The rapidly developing human infant is in high demand for nitrogen as well, sketching a vital and delicate balance between host and gut microbiota.

Many gut symbionts can incorporate ammonium (NH_4_) since most gut bacteria possess the capability to produce a glutamate dehydrogenase and use ammonium in their biosynthetic pathways. Not surprisingly, this usage of ammonium has proven to be one of the most dominant microbial pathways expressed in the infant gut microbiome.^2^ This is important for detoxification, since high levels of ammonium can be detrimental to both microbiota and host.^24^ It can therefore be hypothesized that this is the most common source of nitrogen available in the gut and that microbial metabolism centers around ammonium.^25^ Ammonium itself is interestingly enough only present in trace amounts compared to other HMN sources.^26^ We suspect that the infant gut microbiota catabolizes other nitrogenous compounds to acquire and share ammonium.

This review focuses on microbiome interactions with dietary nitrogen in the infant gut, during the first months of life, particularly with HMN sources. However, infant absorption of many of these components should be considered and is currently elusive. It is unknown exactly how much of the tailored non-protein nitrogen content reaches the colon and is accessible to the microbiota. However, since HM provides such a tailored nutrient input, hypotheses can be formed on how nitrogen is salvaged by the microbiome. A surge in expressional and metaproteomic studies provides information about which bacteria are actively metabolizing in the infant gut. This data contributes to our understanding about which bacterial metabolic pathways are active in early life. This is especially relevant since a functional characterization of nitrogen metabolism is often lacking, while its importance for understanding the gut microbiota has been suggested.^27^ By linking the bacterial genomes, microbiomes *in vitro* findings of active symbionts to dietary input, new avenues for scientific experimentation can be found. Later, these findings can be used to directly link metabolism surrounding HMN to infant health and development.^28^ The nitrogen sources considered in this review are the mother’s secretory waste products, free AAs, polyamines, nitrate and nitric oxide (NO) and nitrogen from HMOs which are all featured in HM.

Sizeable efforts have been made to describe the composition of infants’ gut microbiota through 16S rRNA sequencing,^2,5,29^ and later through functional profiling with metagenomics, metaproteomics and metatranscriptomics.^2,30–33^ Potential functions and their relative occurrence have led to theories on what the microbiota is doing in early life. Furthermore, descriptive studies of infant gut bacteria are included to describe the potential of highlighted bacteria to interact with HMN sources. Combined, the included studies provide information on the bacterial genera *Bifidobacterium*, *Enterococcus*, *Escherichia*, *Bacteroides, Enterobacter* and *Streptococcus* and on their interactions with HMN during the first 6 months.

**Figure 1.**
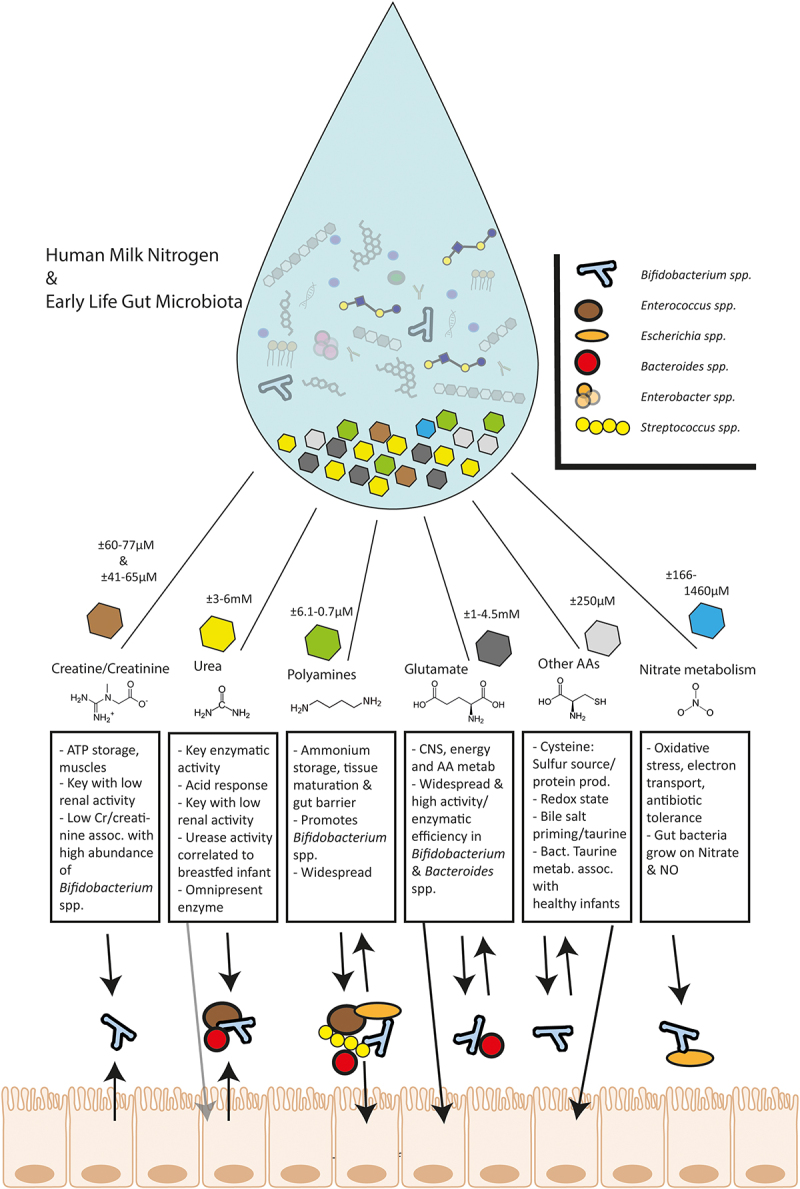
A representation of the important bacterial processes surrounding human milk nitrogen in the colon (HMN) and which bacterial genera are involved, concerning 6 nitrogen sources: Urea, Creatine (Cr)/Creatinine, Polyamines, Glutamate/Glutamine, Other Amino Acids (AAs), Nitrate. Absorption of components by host likely occurs in the small intestine. Hexagons represent relative abundance of components in HM. Abbreviations: assoc. = associated; corr. = correlated; metab. = metabolism; prod. = production; antib. = antibiotic.

## Breast milk derived secretory nitrogen-containing waste products

Secretory waste products, such as urea, creatinine and creatine, deriving from the mother’s metabolism end up in the breast milk.^14,15,17^ Currently, there is no known biological reason for the occurrence of urea, creatinine and creatine in HM. However, gene content related to nitrogen metabolism of the infant gut microbiota has been shown to be responsive to diet.^27,34^ If a supportive microbiome is dependent on receiving these compounds through breast milk, related nitrogen metabolism could prove crucial to our understanding of the early life colonization of the microbiome, especially considering the sensitive development of the gut microbiota during infancy.

### The potential role of urea in the early life microbiota

Urea is the most abundant non-protein nitrogen compound in HM. Urea constitutes up to 15% of total nitrogen in HM.^15,18,22,35^ The human body is incapable of degrading urea through endogenous enzyme production. Urea is a product of human liver metabolism that constitutes of two amine groups connected by a carbonyl group and it is for the largest part secreted through urine production. In lesser extent, urea is secreted in human milk and in the gut. Bacterial Urea Nitrogen Salvation (UNS) has often been suggested as being important for infant gut nitrogen cycling.^15,34,36,37^ Urease (EC3.5.1.5) is the first bacterial enzyme ever described.^38^ It is widely spread across the bacterial kingdom and is especially known as a virulence factor for human pathogens. Regulation and activation of this enzyme have been studied in those pathogens. For example, nickel chelation is required for enzyme activation when studying urease activity in pathogen model systems like *Helicobacter pylori* and *Klebsiella aerogenes*. ^39,40^ On the contrary, urease has been indicated as a health-associated factor.^41^ As the input metabolite, urea can derive from bacterial metabolism due to bacterial arginase activity.^42^ In *Bifidobacterium infantis* (*B. infantis*) it seems that urease activity is limited to the presence of urea.^34^ Urease is involved in microbial amino acid metabolism, yet has also been indicated as a pH regulator as part of an acid response, for intestinal bacteria.^43–45^ This is especially important, since the healthy range of pH in the infant colon is considered between 4.5 and 5.5 with *B. infantis* present.^46^

Bacteria removing urea from the infant gut can be crucial to infant health since liver and kidney systems are underdeveloped compared to adult life.^47,48^ In an adult human host, health status determines the availability of urea in the gut and that subsequently increases urease activity.^41,49,50^ Clearly, urea is also available to the bacterial infant gut colonizers during early life, of which human milk is the major source. Besides originating in breast milk, it is likely to also be secreted into the gut lumen by the neonatal host, as with adults. Notably, in the early days of infant gut studies, it was shown that the urea fecal output is lower than the estimated input through breastfeeding, indicating urea processing in the gut. Furthermore, 15N isotope studies in humans showed assimilation of urea into AAs.^51,52^ Interestingly, microbial nickel transporters were found to be abundant during early life,^33^ which are required as a cofactor for urease enzyme activation. Notably, nickel is found in HM at levels between 1.0 and 51.0 μg/L across studies.^53^ Furthermore, there is evidence that the potential of urease activity relates to the microbiome of breastfed infants. As such, urease genes and the enzymatic activity are more abundant in exclusively breastfed infants.^5,35^

Interestingly, urease activity is linked to amino acid metabolism and thus the acquisition of nitrogen for synthesis in bacterial gut species. Specifically, urease activity was shown in *Streptococcus thermophilus* to be associated with amino acid synthesis and cell growth.^54,55^ Furthermore, *Bifidobacterium* spp. have shown to be urease active.^34,56^ However, it is also clear that not all *Bifidobacterium* are able to access urea as nitrogen source. Urease-active bifidobacteria might, however, prevent pathogens from utilizing this urea. The opportunistic pathogen group of *Enterococcus* spp. have been associated with luminal urease activity, for example.^57^ Notably, *Enterococcus* spp. colonization of the infant gut negatively correlates with infant health status.^32^ *Enterobacteriaceae* is an interesting group when considering urease activity,^58^ as well as *Escherichia* spp. that occur in the early life human gut.^59,60^ Finally, *Bacteroides* can be the genus that profits from urea in mature HM. For example, *Bacteroides koreensis* sp. nov. and *Bacteroides kribbi* sp. nov., two new members of the genus *Bacteroides*.^61^ Importantly, urease activity might be a way to survive in an acidic environment when fatty acid production is high. How nitrogen is further cycled by bacteria is still unclear, although the resulting ammonium should be a suitable nitrogen source for many other microbes in the environment. Thus, both beneficial bacteria and potentially infectious or harmful bacteria are potentially competing over breast milk urea. This competition over urea with commensals prevailing might be crucial for a healthy microbiome.

### The potential role of creatinine in the early life microbiota

Both creatine and creatinine are transported into HM. Measured creatinine concentrations in HM vary greatly, but it was early established in the ±41–65 µM range.^62^ Infant formula often contains higher levels of creatinine, due to its origins in cow’s milk.^62,63^ Creatinine originates in a human host as a secretory product of muscle use and repair.^64,65^ It is stored as phosphocreatine locally, to provide plenty of phosphate for human energy metabolism and specifically ATP.^66^ It is most abundant in skeletal muscle as a reservoir for active outbursts by the host. During muscle metabolism, creatine turns into creatinine in an endogenously irreversible reaction. This creatinine will then be secreted from the host and functions as a biomarker in urine, blood and human milk. Clearing breast milk creatinine by the early life gut microbiome could impact neonatal gut health.

**Figure 2.**
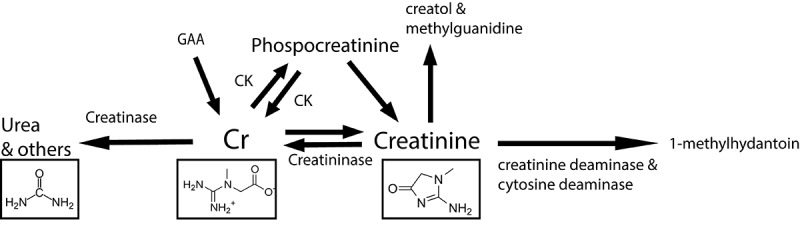
A representation of creatine and creatinine metabolism with pathways important for the infant gut as described above; Abbreviations: creatine (Cr), creatine kinase (CK), guanidinoacetate (GAA).

During life, creatinine will likely be available to the gut microbiome, especially during periods of low kidney functioning. Just as during early life, when kidney and other secretive functions of the human body are hindered or still in development.^47,67–70^ However, it is unclear how much creatinine is secreted in the infant gut by the host. Creatinine degradation occurs through bacterial creatininase activity wherever creatinine is available.^64,71,72^ This occurs via three routes: 1) a combined effort of creatinine iminohydrolase (creatinine deaminase; EC 3.5.4.21) and a cytosine aminohydrolase (cytosine deaminase; EC 3.5.4.1); 2) creatinine amidohydrolase (creatininase; EC 3.5.2.10); 3) and finally a less characterized route via creatol and methylguanidine (Figure 2).^64^ A major product of creatinine degradation via creatinine deaminase is 1-methylhydantoin, an intrinsic hydroxyl radical scavenger or antioxidant.^73,74^ The use of^13−^C-labeling to prove that creatinine is oxidized by mammals into creatol and 5-hydroxy-1-methylhydantoin.^74^ Specific genes involved in this metabolism are hardly characterized by common infant gut symbionts. However, the infant gut microbiota gets a chance of interacting with this nitrogen source in early life.

For every HMN source, we studied its potential in selecting for a health-promoting microbiota. Creatinine is negatively correlated with the abundance of *Bifidobacterium* spp. during the first month of life, indicating a role for the genus in a detoxing effect.^75^ This was confirmed when applying a mixture of *Bifidobacterium* spp. and *Lactobacillus* spp. led to lower creatinine levels in broiler chickens, although the opposite has also been shown in broiler chickens.^76,77^ Therefore, the fate of creatinine in infant gut nitrogen cycling remains elusive. A possible explanation for the conflicting observations can lie in microbiota composition and activity. Perhaps it diffuses into the intestinal tract, to be degraded by the gut microbiome or to be secreted through urine. In the gut, through bacterial metabolism more creatine can become available for the bacteria, so creatine should be considered a potentially important part of HMN.

Creatine (Cr) is provided by HM in concentrations around 77 µM, while others established it at slightly lower concentration.^62,78^ In HM, there was no observed difference between Cr concentrations at 1–2 week postpartum and 5–6 weeks. Interestingly, the formula seems to hold higher concentrations of creatine (>4×), while others are almost deprived of it.^78^ The milk source of these formulas is explaining these levels, with, for example, cow’s milk being rich in Cr. Notably, guanidinoacetate (GAA) is the precursor of Cr in the human body. Edison *et al*. 2013 established, however, that HM is not a clear route of supplying infants with GAA, since a clear presence is lacking.^78^ Interestingly, calculated from intake and an estimated size of the Cr pool in infants, 90% of creatine should originate in endogenous production from energy metabolism and muscle use, which potentially cycles into the intestine.

Creatine functions in bioenergetics for neurological and muscle cells by maintaining ATP levels.^64,79^ A Cr pool exists in the human body to maintain homeostasis in this concern, together with creatine phosphate that can accumulate up to approximately 100 g.^62,64^ As mentioned, creatine is linked to the availability of creatinine as well. Creatine kinase (EC 2.3.7.2) transforms Cr into phosphocreatine, a crucial step in human body energy homeostasis. Creatine kinase is , traditionally, an indicator of health^80^ and that might be impacted by bacterial Cr metabolism in the neonatal gut. Furthermore, there are a few indications that high Cr is associated with infant fed formula.^81,82^ Bacteria that are capable of degrading Cr in the human gut have been identified.^83^ First indications of association of creatine with microbiome composition also exist.^84^ It was shown that Cr negatively correlates with *Bifidobacterium* spp. and positively correlates with *Klebsiella* spp., further indicating the potential for a microbiome dominated by bifidobacteria to have a higher potential for clearing Cr.^85^ Furthermore, probiotic supplementation of *Bifidobacterium lactis* has been shown to alter Cr amounts in rats indicating an improved energy metabolism for the host.^86^ Finally, in the aging mice gut, Cr availability led to an increase in creatine degradation occurrence in the functional profile of the gut.^87^

## Polyamines and their impact on the infant gut microbiota

Polyamines (PAs) are, under physiological pH, polycationic substances, rich in nitrogen that function in human cell growth and tissue maturation. These biogenic amines can therefore be in high demand for rapidly growing tissues, like the developing gastrointestinal tract of the newborn.^21,88,89^ Moreover, PAs are suggested to be involved in immune system maturation, functioning and modulation of gut permeability.^90–93^ In HM, occurring PAs are spermine, spermidine, putrescine and cadaverine all have two or more amino groups.^94^ PAs can derive from either host and bacterial metabolism or diet. The fact that PAs are rich in nitrogen makes it an interesting nutrient source for the colonizing microbiome. In HM, polyamine concentrations increase postpartum^19,20,95^ with concentrations collapsing from the second month onwards.^19,21,96^ Gòmez-Gallego *et al*. investigated polyamine concentrations in HM of healthy mothers. The most abundant was spermine (6.1 µM), followed by spermidine (4.2 µM) and finally, in lesser amounts, putrescine (0.7 µM), with slight variation due to geographic locations in spermidine and putrescine. It was, however, established early that concentrations vary greatly between mothers.^19^ Buts *et al*. (1995) established a similar polyamine profile in HM samples (spermine, 3.1 µM; spermidine, 2.2 µM; and putrescine, 0.24 µM) with a total polyamine concentration of 5.57 µM (±0.18 µM).^97^

The availability of polyamines in the infant colon is currently enigmatic. According to current knowledge, most PAs are, however, absorbed in the upper intestine in support of growth processes of the human body.^98^ In the infant GI-tract, however, optimal adsorption might be developing in combination with a large supply through HM, causing polyamines to reach the colon. PAs occur in the colon of healthy adults within the range of 0.5 to 1 mM.^99^ There, PAs are adsorbed into the human body through the colonic mucosa.^100^ In the colon, bacteria are suggested to be largely responsible for the PAs present there. Bacteria can produce a wide range of PAs, including spermidine, homospermidine, sym-homospermidine, norspermidine, putrescine, cadaverine and 1,3-diaminopropane, while there are also bacteria that are not able to produce any.^101,102^

Synthesis of polyamines is mainly regulated and activated by ornithine carboxylases.^103^ These carboxylases catalyze the decarboxylation of ornithine to produce putrescine. Followed by several methods of elongation, for example spermidine synthase (EC 2.5.1.16), while in the meantime adding one or two more amine groups originating from other nitrogenous compounds (Figure 1). For a long time, biosynthesis of polyamines was mainly studied in *Escherichia coli* (*E. coli*) of which related spp. also occur in the infant gut, albeit as a minor component.^29^ More common microbiome members in the first 2 months of life, e.g. *Streptococcus* spp. and *Enterococcus* spp. have been shown to produce polyamines from AAs.^104^ This enzyme is broadly studied in *Bacteroides* spp.^105,106^ More recently, activity of a carboxyspermidine decarboxylase (*casdc*) was described in *Bacteroides thetaiotaomicron*, a representative of a genus highly present in many life stages, including infancy. This *Bacteroides* strain produces the polyamine spermidine in a polyamine free medium and moreover this that activity provided a growth benefit for the strain under these conditions.^107^ This provides an indication of polyamine metabolism potentially selecting for bacteria in a complex microbial environment. In HM, however, the longer polyamines are more dominant than its precursors (Figure 1).

Polyamines might also provide a health benefit through gut microbiome modulation, and first evidence has been provided.^108^ In mice, supplementation of PAs in formula led to a significant increase of *Bifidobacterium* spp., *Bacteroides* spp., *Clostridium* spp. and *Verrucomicrobia spp*. (as “Akkermansia-like bacteria”) in the large intestine. *Bifidobacterium* spp. were even higher than breastfed pup-mice. Interestingly, polyamine supplementation led to the promotion of autophagy in human cell lines, an indicator of gut health which inhibited the propagation of SARS-CoV-2.^109^ Since autophagy plays a role in gut barrier maintenance, polyamine as a part of our diet can have a health impact.^110^

Here, we summarize the understanding of polyamine metabolism by infant gut bacteria. There is no current evidence of extracellular degradation of polyamines by gut bacteria. However, within the *Bifidobacterium* genus the capability to synthesize and transport polyamines to the intracellular environment has been reported^111^. With spermidine *in vitro*, spermidine is absorbed by the bacteria and in some cases is processed (*Bifidobacterium scardovii*).^111^ No known homologs were detected, indicating a possibility for novel methods of polyamine processing in *Bifidobacterium* spp. Spermine, the longest naturally occurring polyamine in HM, was taken up by more *Bifidobacterium* strains in the study by Sugiyama *et al*. ^111^ Again, the system of choice for this type of transport, a *potRABCD* transport system, was not found in the genus.^111,112^ Moreover, extracellular concentrations of spermine were increasing during growth phase of, among others, *B. infantis*. Also, *Bifidobacteirum adolescentis* (*B. adolescentis*) was proven to export spermidine into the supernatant *in vitro*. ^104^The production of putrescine from ornithine seems to be prevalent in many species of the *Bacteroides* genus. For example, *Bacteroides fragilis* has been noted as possessing this capability with the goal to produce y-aminobutyric acid (GABA).^113,114^ Most studies have shown that cultural conditions tend to be highly specific for strains, leading to low replicability. However, this is a promising mechanism by which the infant gut microbiota promotes health during early life. Especially, since initial evidence showed that polyamine supplementation in formula counteracts allergy occurrence and gut permeability issues.^93^ However, bacterial metabolism concerning polyamines has hardly been considered to play a role in infant health. According to the literature reviewed here, polyamines are bifidogenic. It makes this part of HMN interesting for future study.

## AA metabolism & the GABA shunt

Both bacterial protein fermentation and human digestion can lead to the liberation of amino acids (AAs). Before that, proteases produced by the mother digest human milk protein.^115^ This review highlights that the commonly released AAs are at the core of bacterial metabolism and their survival.^116^ Where there are AAs, bacteria multiply. This review proposes that the infant gut is not any different. If the first meals to pass through the newborn’s GI-tract are developed to kick-start bacterial metabolism, AAs might be key.

## The role of glutamate, glutamine for the infant gut microbiota

The amino acid glutamate, however nutritionally nonessential, plays a role in many important metabolic processes, including the citric acid cycle, protein synthesis and acts furthermore as a precursor for several bioactive compounds.^117,118^ Glutamate is the most dominantly occurring free amino acid in HM, only closed by its close relative glutamine. Glutamate as a dominant dispensable free amino acid occurs in the range of 960.1–1529.0 µM, reaching peak supply at 4 months into the lactational period. However, a higher concentration of 4.5 mM has been argued.^6,14,119,120^ Free glutamate is during this period proportional to the amount of glutamate supplied through protein, in which glutamate is also the most dominant AA.^121^ This level of free glutamate is significantly higher than the level found in the average cow’s milk-based formula.^120,122^ Interestingly, it is also higher than the 30 mg/kg BW (0.204 mmol/kg BW) per day acceptable daily intake (ADI) of free glutamate set by the European Food Safety Authority for infants, due to neurotoxicity concerns. An infant weighing 5 kg receiving a recommended 0.75 L of human milk would, according to that standard, only receive 0,76 mmol (1,02 mM) of free glutamate. Breastfeeding being the infant feeding method of choice makes it likely potential health benefits underly the presence of these levels of glutamate. Bacterial interaction in the infant gut with glutamate is likely a very prominent part of infant gut nitrogen cycling.

**Figure 3.**
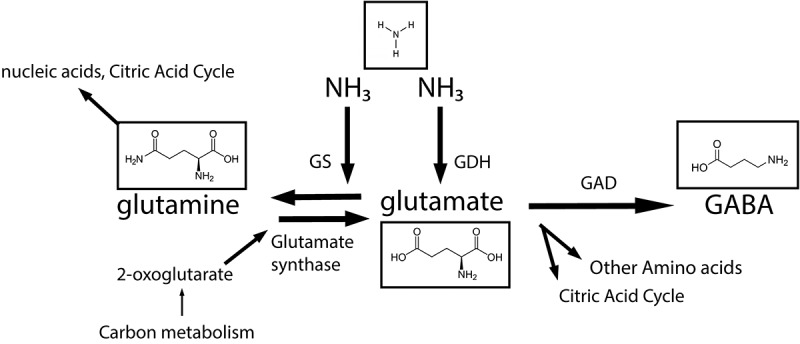
A representation of glutamate metabolism by both bacteria and the human host as an important nitrogen cycle for the early life gut. Abbreviations: glutamine synthetase (GS), glutamate dehydrogenase (GDH), glutamate decarboxylase (GAD), γ-aminobutyrate (GABA).

Glutamate catabolism is achieved through glutamate dehydrogenase (GDH) or glutamate decarboxylase (GAD, Figure 3). The GDH enzyme leads to the assimilation of ammonia into AAs, with glutamate as the starting point. Glutamine synthetase (GS) catalyses the reaction of glutamate to glutamine.^117,123^ Glutamate metabolism can be part of bacterial stress responses, including acid responses.^118^ Glutamate decarboxylase is involved in acid stress response, causing decarboxylation of glutamate to γ-aminobutyrate (GABA). GABA is the main inhibitory neurotransmitter, while glutamate, its precursor, is the main excitatory neurotransmitter.^124,125^ Bacteria can then export it through a GadT2 Glutamate/GABA antiporter, after which is absorbed into the human body, providing benefits for health and development in the nervous system.^126,127^ Notably, the human body can convert glutamate through GAD-activity, hence the high demand of glutamate for the infant body and its developing nervous system. Interestingly, a ferredoxin dependency occurs in the GABA-shunt via dependency of the glutamate synthase.^128^ This enzyme catalyses the production of glutamate from 2-oxoglutarate and glutamine as nitrogen source. However, ferredoxin metabolism is not commonly studied in the infant gut. Physiologically, an external supply of glutamate could replace a need for endemic production of this amino acid. In the developing human body glutamate serves a wide array of purposes in neurology and energy homeostasis. Bacteria-wise, a metaproteomics study by Xiong *et al*. showed that among conserved functional groups was glutamate dehydrogenase, in all studied infants.^30^ The importance of glutamate is furthermore indicated by the fact that it is almost completely metabolized first pass in infant pigs. Such a scenario is likely in the human infant gut as well.^129^

There is evidence for glutamate metabolism by bifidobacteria.^130^ All included Bifidobacterium strains (*Bifidobacterium breve*, *Bifidobacterium longum* subspp, *Bifidobacterium pseudolongum*, *B. adolescentis*) showed both synthethase and dehydrogenase activity for all, although at different levels.^130^ Interestingly, lower Km values for the glutamate synthetase of *Bifidobacterium* spp. compared to almost every other included strain except a *Lactobacillus* sp., indicating that *Bifidobacterium* is an efficient genus in detoxifying the infant gut from ammonia. Indicating a key role for *Bifidobacterium* spp. in glutamate cycling, the purified GS of *B. bifidum* reacted optimally in an acidic environment, which was not the case for the non-*Bifidobacterium* spp.^131^ This would match the infant gut’s acidic conditions. Glutamine and glutamate cycling by *Enterococcus* and *Streptococcus* spp. has been studied, but in the small intestine of ruminants or in the bird gut.^132,133^ Finally, the production of GABA from glutamate as an important metabolic route should be investigated. *E. coli* was one of the organisms in which it was shown that GABA metabolism conferred acid resistance in bacteria.^134^ Recently, The production of GABA has been contributed to more common infant gut symbionts like *Bacteroides* spp.^114^

## Other predominant amino acids: Taurine and Cysteine

Taurine or aminoethylsulfonate (a C2 sulfonate) is a sulfur-containing amino acid in HM. Taurine is the third most dominant-free amino acid in HM.^16,17,135,136^ Moreover, the infant’s body can synthesize taurine but not degrade it, making plenty of the amino acid available for microorganisms. Namely, a large portion of taurine is secreted from the human body in the form of taurine-conjugated bile salts.^137^ This requires the release of taurine by bile salt hydrolases by bacteria. One of those genera is *Bacteroides*.^138,139^ Fermentation of taurine is dependent on the cleavage of the inert sulfonate C-S bond.^140^ A process not commonly described for anaerobic human gut bacteria, even though it is closely linked to the production of H_2_S, toxic to the human body. Moreover, when it does occur, like *e.g* in *E. coli*, it concerns a process involved in aerobic growth.^141,142^ There is a diverse range of strategies for cleaving the C-S bond, yet not all are anaerobically feasible. Among the known pathways is a thiamine pyrophosphate-dependent sulfoacetaldehyde acetyltransferase system (Xsc) which occurs under both aerobic and anaerobic conditions.^140,143^ Further anoxic options are still poorly understood. One of the more remarkable is the description of an IseG-dependent system, where also L-alanine can be produced, in opportunistic pathogen *Bilophila wadsworthia* and *Desulfovibrio piger*. ^143^ Collard *et al*. went further and described taurine and its related physiological processes as key contribution to resistance to new infections.^144^ In another study, taurine alone did not alter immune responses in the lamina propria.^145^ However, it did affect gene expression in epithelial cells and, more interestingly, it showed the importance of a taurine trained microbiota. That led to a greater systemic resistance against *Klebsiella pneumoniae* and lower oxygen availability.^145^ The relation to oxygen makes it so that the lack of a taurine-trained microbiota could lead to more potential for opportunists to colonize, like *Enterococcus* spp., a common pediatric infection source in the neonatal period. In another study, a healthy infant control group showed significant higher levels of taurine metabolism through a metagenomic approach, confirming a role in the healthy infant’s metabolism.^146–148^ Targeting of taurine utilizers can benefit options for infant health care and nutrition.

Cysteine is another amino acid in HM that is potentially important to the microbiome. The biosynthesis of the amino acid cysteine serves to incorporate inorganic sulfur into organic matter.^149^ Cysteine then plays a crucial role in many catalytic sites of subsequent protein and protein folding by being part of disulfide bonds.^150^ Interestingly, bifidobacteria as dominant colonizers of the gut seem to be auxotrophic for the amino acid.^151^ *B. bifidum* (multiple strains) has shown reduced growth only when cysteine is lacking in an evaluation of auxotrophy across AAs occurring in HM.^151^ Cysteine metabolism has also been indicated as a key metabolite that inhibits gut-related oxidative stress.^152,153^ Conclusively, both these AAs need to be considered in relation to infant feeding and the infant gut microbiota (Figure 1).

## Breast milk derived nitric oxide, nitrate & nitrite

Nitric Oxide (NO) is involved in physiological processes in the gut that can determine an individual’s health.^27,154,155^ Nitric oxide is synthesized from L-arginine by a nitric oxide synthase (NOS) and is involved in vasodilation, neurotransmission, the immune system, gene expression and regulation. Notably, NO is involved in triggering of lactation and might, for that reason, be featured in HM.^156^ Notably, NO occurs in HM and concentrations peak in the first week postpartum.^157^ Furthermore, it seems to be important for the oxidant and antioxidant status of human breast milk during lactation period.^158^ The role of NO for the microbiota is currently elusive, but it might have a function that is lacking from the gut of infants fed formula products.^159^ Interestingly, NO is involved in interactions between bacterium and host.^160^ However, NO synthesis by bacteria in the infant gut, which handles oxidative stress, electron transport or antibiotic tolerance by the strains, can potentially be harmful as well.^160^

In healthy adults, 1/3 of dietary nitrate ends up in the lower intestine, but only up to 1% ends up in the feces.^161^ Nitrate can provide a growth advantage for strains belonging to the genera *Escherichia*, *Bifidobacterium* spp. and *Lactobacillus* spp. under anaerobic conditions and low oxygen conditions (Figure 1).^162^ Furthermore, nitrate is an electron acceptor when oxygen is limiting, a realistic scenario in the infant gut a few week postpartum.^162–165^ *E. coli* even possesses three nitrate reductases that are active under anaerobic conditions.^162,166^ Nitrite can be toxic at higher concentrations and is therefore excreted to the environment. Interestingly, a study by Tiso & Schechter showed that *in vitro* culturing of infant gut symbionts produces large amounts of fatty acids and the subsequent acidification drives nitrite disproportionation to NO.^162^ To our current knowledge, *Bifidobacterium* spp. do not possess enzymes to produce NO from nitrite. However, an environment dominated by *Bifidobacterium* spp. could theoretically produce levels of NO that affect gut health and integrity because of their role in the acidification of the environment.

## The role of Nitrogen from Human Milk Oligosaccharides and other glycoconjugates for the infant gut microbiome

Human Milk Oligosaccharides (HMOs) are indigestible carbohydrate structures in amount and complexity unique to the milk composition of us humans. HMOs are the third most dominant carbohydrate source (after lactose and fat) available to the microbiome, occurring as high as 15 g/L.^167,168^ Especially so, since the infant is incapable of degrading these complex polysaccharides and only small amounts will be absorbed intact and thus they will reach the colon largely unscathed.^169,170^ So far, proven prebiotics such as galacto-oligosaccharides (GOS) and fructo-oligosaccharides (FOS) have been used in infant nutrition. In recent years, the first synthetic HMO structures are available. The oligosaccharides are the main prebiotics used to enforce formula products, so that they better promote *Bifidobacterium* spp.^10,170–172^ HMOs, besides being a major carbon source for the infant gut microbiota, were shown as a nitrogen for those same bacteria.^173^ The simplest HMOs are derivatized lactoses such as galactosyllactoses and fucosyl and sialyllactoses. The usual composition of detected HMOs follows the formula Lx/y-z (with L: lactose; x: Gal-GlcNac disaccharide units; y: fucoses and z: sialic acids).^174^

Nitrogen in HMOs is provided via (*N*-Acetylglucosamine) GlcNac and sialic acid.^175,176^ Neutral HMOs following this formula were detected between 8 kDa and 10 kDa which means that the majority of HMO-structures contains nitrogen with GlcNAc. It was also determined that acidic HMOs of 3.5 kDa and higher provide additional nitrogen from sialic acids beyond the core GlcNAc nitrogens.^177,178^ On a different resolution, the HMO lacto-N-tetraose (LNT) and lacto-N-neotetraose (LNnT) contain this nitrogen, which are, respectively, featured in Type I and Type II HMOs.^167^ The classification of the types is dependent on the different linkages of the GlcNAc to the galactose. Namely, β1–3 or β1–4 linkages, for Types I and II, respectively.^179^ Notably, for some bacterial species, GlcNac is even a strict requirement.^180^ GlcNAc nitrogen is likely utilizable by more infant gut colonizers in the early stages of infancy. N-Acetylneuraminic acid (Neu5Ac) nitrogen can also prove to be a key nitrogen source as it is the predominant sialic acid in HM.^175,181^ Furthermore, HM is not only rich in HMO glycoconjugates, since other components in HM are rich in glycosylation. These can exhibit certain biological functions, digestive survival and serve, just like the indigenous host mucins,as substrates for saccharolytic bacteria. In particular, glycolipids and glycoproteins need to be considered.^182–184^

Several bacteria thriving in the breastfed infant’s gut have been shown to degrade HMOs or parts of them. This activity is thus also potentially liberating some nitrogen sources, although that specific focus is rare. They are specifically interesting because there are strong indications that HMOs reach the infant gut largely undigested. Upper-small intestinal enzymes do not have a significant impact on the HMO structure.^169^ James *et al*. established that *Bifidobacterium breve* (*B. breve*), among many *Bifidobacterium* spp., holds the capability to degrade LNT, LNnT via different pathways.^167^ HMO utilization by *Enterobacteriaceae* has also been studied.^185^ The study showed that none of the *Enterobacteriaceae* strains grow on 6-siallylactose (6-SL) and LNnT. Although the potential to interact with LNnT for some infant gut bacteria is apparent, the role and effect of the included nitrogen on bacterial metabolism is clearly under established. Meanwhile, there are several bacterial species from the genus *Bacteroides* and probiotic *Akkermansia muciniphila* that have been shown to interact with LNT and LnNT.^186,187^ Since these bacteria, specifically, are promoted by breastfeeding, the relationship between the unique structure of HMOs and their prevalence is confirmed. The obvious suggestion is that HMOs are a preferred substrate for *Bifidobacterium*, meanwhile making the nutrients and the nitrogen less available for less beneficial bacteria. The bonus of having almost exclusive access to nitrogen embedded in HMOs should not be underestimated for bacteria in an increasingly competitive infant gut.

## Discussion

Although this research field is in its early stages, plenty of evidence shows that from the early onset of life, the gut microbiota is involved in catabolic and synthetic activities involving HMN. The neonatal microbiota is highly susceptible to outside influences, like the diet. This makes the early life gut microbiota a very suitable platform to study the impact of nitrogen on the microbiota and on subsequent health. This review focused on the relationship between the settling infant gut microbiota and the non-protein part of HMN. Moreover, it is becoming clear that the human host stands to benefit from this early life bacterial nitrogen cycling. We can also conclude that more data is needed, quantitatively and qualitatively, *in vivo* and *in vitro*, on an -omics scale, and through an *in-vitro* approach with early life microbiota members. Metabolomic, (meta-)proteomic and metagenomic data from clinical studies can help elucidate what the bacteria do in the infant gut, while breastfeeding ensues. For example, ever since the first attempt by Klaassens *et al*. to use metaproteomics to functionally characterize the infant gut microbiota,^188^ meta-studies have become increasingly effective at describing bacterial activity in the gut. On the other hand, studying how important (*Bifidobacterium*) species react *in vitro* to HMN can provide evidence as well.^34,186^ This review provides further evidence on the fact that many aspects of breastfeeding are tailored to suit the infant’s and gut microbiota’s early life needs. The many relations between HMN and bifidobacteria can explain why the genus is successful in the infant gut.

For some nitrogen sources, bacterial species seem to have somewhat exclusive access. While for others, like urea, many of the early life symbionts of the breastfed infant have the capability to process it. As the main non-protein nitrogen source in HM, this component might prove a key metabolite in establishing the early life microbiota. The knowledge of specific bacteria degrading and processing creatinine, creatine, or polyamines is far more elusive. For example, for creatine and creatinine metabolism genes are hardly found or described in the common infant gut symbionts. Nonetheless, both metabolites/nutrients are involved in host health and the impact of these nitrogen sources should be investigated more in depth to explain the role of HM. This will help develop formula products in such a way that they promote a health-inducing microbiota in a similar fashion. As has been hopefully indicated by this review, many of the described nitrogenous compounds can be the result of existing interspecies networks between common infant gut symbionts. Clinical studies specifically focused on dietary nitrogen and infant gut microbiota can therefore elucidate which processes matter the most *in vivo*. In contrast, future *in vitro* studies should determine if and under what conditions these bacteria produce or consume the HMN. Furthermore, future research should focus on determining if nitrogen cycling is part of metabolic interspecies networks, to what extent competition over HMN occurs and if certain interactions exist with human cells in that environment.

There is more nitrogen in HM, besides the non-protein part, there is of course bioactive protein. This review does not understate the potential importance of bioactive protein or other nitrogenous substrates in the early life diet. For example, lactoferrin (LF) is among the most detected proteins in the early life gut, indicating its availability to the microbiota and showing the potential for this (±700 AAs) protein to have a beneficial effect in lowering pathogen colonization.^189,190^ Furthermore, products of the bacterial colonists of the infant gut could prove to be crucial vitamins. Vitamins are at the center of human health and are a product of our diet and bacterial metabolism.^116,191^ HMN could ensure that bacteria are producing vitamins at the right place and at the right time. Is that dependent on the right nitrogen source as input?

In general, the HMN supply seems fit for promoting microbial growth and making sure the gut is colonized in the early life stages. This is accomplished by the presence of GABA-shunt metabolites. The role of these free AAs in HM has once again been confirmed. Namely, much of the early life gut symbionts seem to possess the potential to process glutamine and glutamate, the two main free AAs in HM. The free AAs seem to be there to promote general microbial growth when the gut is still relatively low on microbial mass. This nitrogen is perhaps cycled into vitamins and neurogenic compounds that can have a profound effect on the development of the infant.

Nitrogen input can steer a microbiome in a certain direction and affects the output toward a human host. Direct connections to infant health are hard to establish, however due to the function or risks of certain HMN metabolites we can hypothesize on the importance of the microbes for infant health. This review serves to establish the connections between the microbes and the HM diet and to indicate that plenty concerning nitrogen metabolism is elusive. For example, when looking at the expression levels of a *B. longum* strain in breast milk compared to glucose medium and formula, a nitrogen regulatory protein (N-II EP) *e.g*. is up-regulated,^192^ indicating underlying regulation and response to the presence of HMN. Determination of HMN concentrations that are suitable for infant formulas is difficult due to the lack of consensus of HM concentrations.^21^ Concentrations of HMN sources described are largely dependent on time of day, lactation stage or type, maternal diet, among others.^193,194^ Clearly, the GABA shunt and urea provide a crucial research window in studying infant gut nitrogen cycling and its relationship to bacterial survival and health. The involved metabolites surround one of the most conserved and active metabolic pathways in the infant gut, and its products are crucial for both bacterium and host.

## Conclusive words

We have only just started to unravel the digestive microbial processes that lead to a healthy infant gut. It is clear, however, that much can be learned by studying the interaction between nitrogen and the settling of the infant gut microbiota. Nonetheless, the review indicates the importance of specific HMN-constituents that include urea, glutamate and polyamines for the infant gut microbiota and human health. This contradicts the major premise that there just needs to be dietary nitrogen available through protein. However, there is a lot to be achieved mechanistically describing the importance of the composition of HMN. We have described the occurrence of dominant microbial pathways and the effects of the occurrence of these nitrogen sources on gut microbiota composition from available literature. Combined, this review provides an overview of the current relationship between HMN and the most prevalent gut symbionts during early life.

## Data Availability

Data sharing is not applicable to this article as no new data were created or analyzed in this review.
